# Using Polymeric Carbon Nitride/ZrO_2_ Nanocomposite for Low Salinity Water Flooding in Carbonate Porous Media at Real Reservoir Conditions

**DOI:** 10.3390/polym17050649

**Published:** 2025-02-28

**Authors:** Yaser Ahmadi, Marjan Tanzifi

**Affiliations:** Chemical and Petroleum Engineering Department, Ilam University, Ilam P.O. Box 69315/516, Iran

**Keywords:** low salinity flooding injection, carbonate rocks, spontaneous imbibition, wettability modification, polymeric carbon nitride/ZrO_2_ nanocomposite

## Abstract

Nanoparticles and nanocomposites have been used in recent studies to improve oil reservoir recovery. With the introduction of a newly constructed smart water injection scenario, this work investigated the physicochemical characteristics of the polymeric carbon nitride/ZrO_2_ nanocomposite (ZrO_2_/g-C_3_N_4_), and the results were compared with pure ZrO_2_ nanoparticles as a known enhanced oil recovery agent. The effects of ZrO_2_/g-C_3_N_4_ and ZrO_2_ on the wettability change, zeta potential, and interfacial tension under reservoir conditions (78 °C and 3800 psi) were determined after characterization experiments, which included X-ray powder diffraction (XRD), a Fourier transform infrared spectrometer (FTIR), transmission electron microscopy (TEM), a field emission scanning electron microscope (FESEM), energy-dispersive x-ray testing (EDX), and a Brunauer–Emmett–Teller (BET) analysis. Based on the highest zeta potential and the greatest reduction in the contact angle and interfacial tension, the optimum concentrations for ZrO_2_/g-C_3_N_4_ and ZrO_2_ were determined to be 30 and 40 ppm, respectively. Moreover, the ZrO_2_/g-C_3_N_4_ nanocomposite demonstrated better results in enhancing the oil recovery parameters, and it was selected for low salinity flooding scenarios with three different salinities, including MgCl_2_ + seawater (SW), CaCl_2_ + SW, and MgSO_4_ + SW, at 30 ppm of the nanocomposite. The best readings for the ZrO_2_/g-C_3_N_4_ nanocomposite in its interfacial tension, contact angle, and zeta potential show that 1000 ppm has the best interfacial tension reduction among the tested concentrations of 500–50,000 ppm. At 30 ppm, MgCl_2_ + SW had the maximum recovery (i.e., 49.36%), and this resulted from better interfacial tension reduction, contact angle reduction, and stability compared to other salinities.

## 1. Introduction

**Figure 1:**
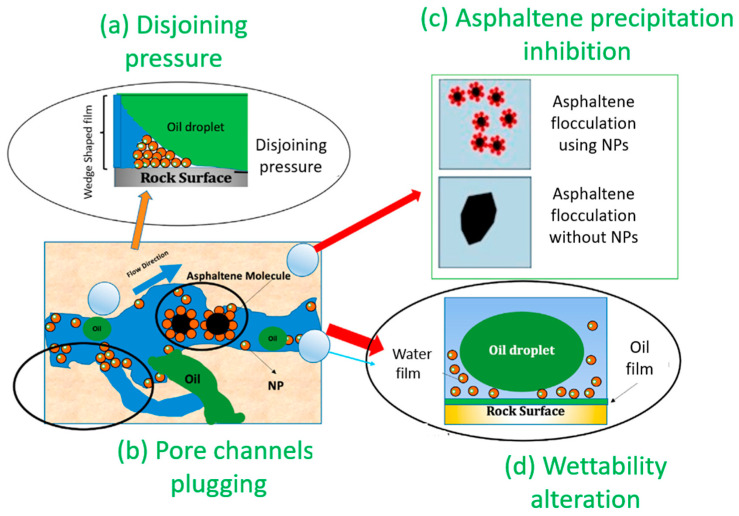
EOR mechanisms using nanoparticles including (**a**) disjoining pressure, (**b**) pore channels plugging, (**c**) asphaltene precipitation inhibition, and (**d**) wettability alteration [21].

Nanoscale materials are used in nanotechnology, which has numerous practical usages [1]. To improve oil recovery, researchers have employed nanoparticles to modify the physical properties of oil and enhance its flow through oil and gas reservoirs [2,3,4]. Nanotechnology is used in conjunction with different novel technological processes to recover more oil compared to routine scenarios [5,6,7,8,9]. Nanoparticles and nanocomposites can enhance oil recovery (EOR) by changing the main mechanisms in the reservoir (porous media), including the contact angle (CA) and interfacial tension (IFT) [10,11,12,13]. When applied to porous media, nanofluid flooding scenarios typically decrease the CA and IFT [14,15,16]. Two phases (in this case, oil/water and oil/nanofluid) are necessary for defining the IFT, and it is the force that holds the surface of a certain phase together. A liquid surface (in this case, oil) and a solid surface (in this case, carbonate sheets) meet at an angle called the CA when an immiscible fluid (in this case, nanofluid) is present. Because nanoparticles are smaller than other particles, they may help keep other substances from clogging the rock pores and throats when they are introduced into reservoirs [17]. The reservoir’s temperature, pressure, salinity, synthesis properties, stability of nanoparticles, and concentration all have an impact on the nanofluid’s efficiency in recovering more oil in the reservoir [18,19]. Because of nanoparticles’ electrical and physicochemical properties, which also allow them to absorb at oil–water interfaces, nanoparticles can play an efficient role in EOR [20]. Figure 1 illustrates the many functions of nanoparticles in EOR situations, such as (a) disjoining pressure, (b) blocking pore channels, (c) inhibiting asphaltene precipitation, and (d) altering wettability [21]. The oil droplets are more mobile within the porous surface and achieve better recovery than the conventional approach, as illustrated in Figure 1, because the nanoparticles self-assemble into a wedge-like film at contact and exert high disjoint pressure due to the increased entropy and electrostatic force of repulsion. Increased structural disjoining pressure from nanoparticles, advantageous rheological control, a decrease in IFT, a change in wettability, and the avoidance of asphaltene precipitation all contribute to the displacement recovery process [22,23].

Zirconium dioxide (ZrO_2_) nanoparticles are regarded as efficient materials in different chemically enhanced oil scenarios [24,25,26,27]. Karimi et al. (2012) examined how ZrO_2_ nanoparticles affected the EOR process. According to their findings, these nanoparticles might transform carbonate rocks from being strongly oil-wet (tending to adsorb oil on the rock surface) to being strongly water-wet (tending to adsorb water on the rock surface) [24]. The ZrO_2_ nanoparticles were used in low salinity flooding (in this type, the selected brine concentration was injected into porous media) in combination with a surfactant by Dordzie et al. (2022) in carbonate fracture porous media. Based on their results, using ZrO_2_ with surfactants was efficient in low salinity water flooding, and the original oil in place in the reservoir was increased to 7% [25].

The combination of nanoparticles/polymers has generated interesting results among different researchers. Mohanty et al. (2021) stated that biopolymers have been employed in EOR due to their high viscosity and their significant effects on waterflooding (injecting water) performance. They used ZrO_2_/Sodium alginate, and it was observed that enhancing the salinity from 0.015 to 0.1 wt % in the 0.04 wt % ZrO_2_ nanoparticle suspension comprising 0.1 wt % sodium alginate showed a progressive decrease in viscosity under the temperature range from 25 to 50 °C. The proposed sodium alginate/zirconium oxide nanoparticles from the mentioned results indicate their potential for enhanced oil recovery applications [26]. Salem et al. (2024) recently used zirconia in combination with partially hydrolyzed polyacrylamide (HPAM) for EOR with measurements of the IFT, viscosity, and oil recovery [27].

Combining solid materials with beneficial nanoscale characteristics, in which each of the materials has a high level of efficiency, was recently used in preparing efficient nanocomposites. Based on this idea, Ahmadi et al. (2021) used a ZrO_2_/zeolite/cerium nanocomposite for changing the EOR parameters during the water alternating gas injection in the reservoir, and based on their results, introducing ZrO_2_ increased the specific area of the nanocomposite, and this phenomenon was efficient in changing the IFT, CA, and recovery factor. At 100 ppm of the prepared nanocomposite, the recovery was increased from 42.5 to 72% [28]. To enhance the specific surface area of the pure ZrO_2_, we synthesized two-dimensional nanosheets with a network of numerous and inherently homogeneous pores by combining zirconium with graphitic carbon nitride (g-C_3_N_4_). These materials are simple to synthesize and inexpensive, and they were presented as potential agents for EOR scenarios.

Recent research indicates that by converting the wettability of reservoir rocks from oil-wet (OW) to water-wet (WW), the oil recovery increases using low salinity water flooding (LSWF) [29,30,31,32]. LSWF performs better than other EOR techniques in terms of affordability, ease of use, and sustainability [33,34,35,36,37]. Based on the previously demonstrated applications of ZrO_2_ in effectively changing the EOR, the usage of g-C_3_N_4_ in increasing the specific surface area, and high applications of low salinity water flooding, the main focus of this work was the synthesis of the ZrO_2_/g-C_3_N_4_ nanocomposite for low salinity flooding. The results were then compared with pure ZrO_2_. A range of assays were used to analyze the chemistry, surface, and ZrO_2_ and ZrO_2_/g-C_3_N_4_ nanocomposite structures. The optimum ZrO_2_ and ZrO_2_/g-C_3_N_4_ concentrations were found by applying the contact angle, IFT, and zeta potential tests at various concentrations, such as 10, 20, 30, 40, 50, and 60 ppm. The lowest IFT and CA, and the highest zeta potential (ZP), were used to determine the optimum concentrations. The stability of colloidal dispersions and nanoparticle suspensions is largely determined by the ZP. It calculates the electrical potential difference between the stationary layer of fluid affixed to the scattered particle and the liquid’s bulk. Next, the best optimum concentration results, three CaCl_2_, MgCl_2_, and MgSO_4_ results at the optimal nanocomposite concentrations, were used to obtain the best scenarios for low salinity floods. The IFT measurements were conducted at 500, 1000, 2000, 5000, 10,000, and 50,000 ppm of SW+ (CaCl_2_, MgCl_2_, and MgSO_4_) salt concentrations. Finally, the recovery tests were compared using 1000 ppm (which has the best reading) of SW+ (CaCl_2_, MgCl_2_, and MgSO_4_). This work’s primary innovation is that all of the experimental designs were informed by field conditions. The designed scenarios were novel, and in this study, the best optimum scenarios were suggested for carbonate porous media.

## 2. Materials and Procedures

We chose crude oil and cores from a carbonate oil deposit in western Iran. Oil has a viscosity of 12.45 cP and a density of 0.979 gr/cm^3^ at 78 °C. The porosity, permeability, core length, and core diameter were 13.40%, 10.78 md, 4.80 cm, and 3.65 cm, respectively. As the brine source for all of the planned tests, we used seawater (SW) containing the salts NaCl (2840 ppm), CaCl_2_ (138 ppm), MgCl_2_.6H_2_ (643 ppm), and KCl (80 ppm). A melamine powder (C_3_H_6_N_6_, 99%) was prepared from Sigma-Aldrich (Louis, Canada). Merck (Rahway, NJ, USA) supplied the following: ammonia solution (NH_3_, 25%), zirconyl chloride octahydrate (ZrOCl_2_.8 H_2_O), and ethanol (C_2_H_5_OH).

The ZrO_2_ was synthesized in a typical process; zirconyl chloride octahydrate (3 g) was added to 40 mL of deionized water and mixed quickly for 60 min. Next, the NH_3_ solution was added dropwise into the first solution until the pH reached 10.5. After stirring for 60 min, filtering, and washing with distilled water, the obtained white gel was dried for eight hours at 100 °C. The product was then ground and heated for three hours at 500 °C at a ramp rate of 10 °C per minute [38]. The melamine powder was directly annealed twice to create g-C_3_N_4_ nanosheets. Initially, a predetermined quantity of melamine was placed in a ceramic crucible with a lid and annealed for four hours at 550 °C (ramp rate of 5 °C/min) in a tube furnace. After milling to a powder, the final product (g-C_3_N_4_ bulk) was placed in three open ceramic crucibles and heated for two hours at 500 °C at a ramp rate of 5 °C per minute (g-C_3_N_4_ nanosheet) [39]. Ultimately, ZrO_2_/g-C_3_N_4_ nanocomposites were created using the solution mixing–calcination method. The standard procedure involved dispersing the ZrO_2_ and g-C_3_N_4_ (*w*/*w* 1:1) in ethanol for 60 min and then stirring for 20 h. The final polymeric nanocomposite was obtained by annealing the powder at 400 °C for one hour after it had been dried, milled, and rinsed with an ethanol/water solution.

**Figure 2:**
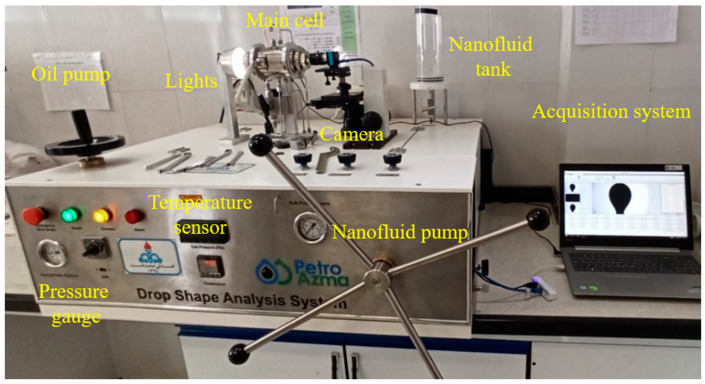
The interfacial tension/contact angle setup using the polymeric nanocomposites. This setup contains the main items, including the main cell, oil and nanofluid pump, temperature and pressure gauges, nanofluid tank, lights, and acquisition and camera system.

The experimental setup for measuring the IFT and CA using carbonate sheets when polymeric nanocomposites are present is depicted in Figure 2. This setup is used for measuring the interfacial tension and contact angles of base (without nanoparticles), ZrO_2_, and polymeric ZrO_2_/g-C_3_N_4_ nanomaterials at different concentrations and review conditions. Measuring the IFT and CA was used for selecting the optimum concentrations of nanoparticles, and it was performed at different concentrations, including 10, 20, 30, 40, 50, and 60 ppm with a base fluid of seawater containing the salts NaCl (2840 ppm), CaCl_2_ (138 ppm), MgCl_2_.6H_2_ (643 ppm), and KCl (80 ppm). First, the base or nanofluids (ZrO_2_ and polymeric ZrO_2_/g-C_3_N_4_) were prepared with a combination of nanoparticles with seawater brine and stirring with ultrasound. Second, the prepared nanofluids were brought to the main cell, and the cell pressure and temperature were allowed to become stable at 3800 psi and 78 °C, respectively. The pressure and temperature were applied with a nanofluid pump and heating jacket, respectively. Third, the oil was injected through a syringe inside the main cell. Finally, the camera captured the oil droplet, which is surrounded by nanofluid in the main cell. Through the analysis of this droplet with the in-house software, Petroazma’s program, the IFT was measured. The contact angle was determined in this instance after a droplet came into contact with the carbonate rocks, which had previously been placed in the main cell, but the procedure was the same as for the IFT. Thus, a layer of carbonate rock was first placed in the main cell, and then a base or nanofluid was injected into the main cell in the presence of the rock, setting the pressure and temperature. Finally, as with the IFT, the oil was injected with a syringe, and by analyzing this droplet in contact with the rock using the in-house software, the contact angle was measured. In this study, the contact angle and the IFT tests were conducted three times, and the average is provided.

**Figure 3:**
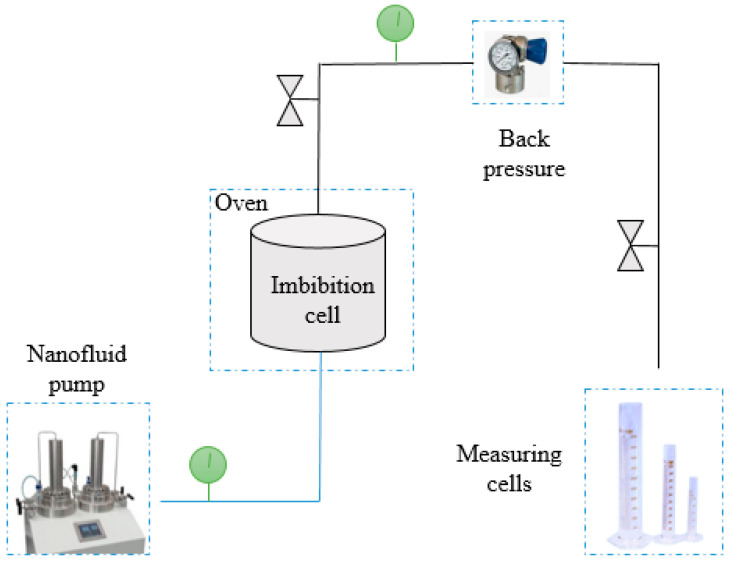
Imbibition apparatus using nanocomposites. Apparatus contains imbibition cell, oven, nanofluid pump, back pressure, and measuring cells.

After selecting the optimum concentrations of nanoparticles, I thoroughly measured the IFT and CA. A series of IFTs and CAs with the same procedure were designed using different brine salinities, including MgCl_2_ + SW, CaCl_2_ + SW, and MgSO_4_ + SW. As the polymeric ZrO_2_/g-C_3_N_4_ had better results than the pure ZrO_2_, it was selected for performing the imbibition tests at the optimum concentration, which was 30 ppm, as the schematics show in Figure 3. It contains different elements, including a main cell, an oven for applying the temperature, a nanofluid pump for setting the pressure, measuring cells for obtaining the oil volumes, and back pressure for maintaining the pressure of the cell at the reservoir pressure. First, the initial core mass, oil-saturated core mass, space volume, and oil saturation were measured before the imbibition trials. Second, four tests were selected for performing the imbibition tests: (a) base fluid (without nanoparticles), (b) MgCl_2_ + SW + 30 ppm ZrO_2_/g-C_3_N_4_, (c) CaCl_2_ + SW + 30 ppm ZrO_2_/g-C_3_N_4_, and (d) MgSO_4_ + SW + 30 ppm ZrO_2_/g-C_3_N_4_. Next, aged core containing crude oil was placed in the imbibition cell and surrounded by nanofluid (a–d scenarios) at 3800 psi and 78 °C. After setting the pressure and temperature, the extraction volume of the oil was monitored with time. The displacement efficiency is obtained by dividing these obtained oil volumes by the core pore volume.

## 3. Result and Discussion

**Figure 4:**
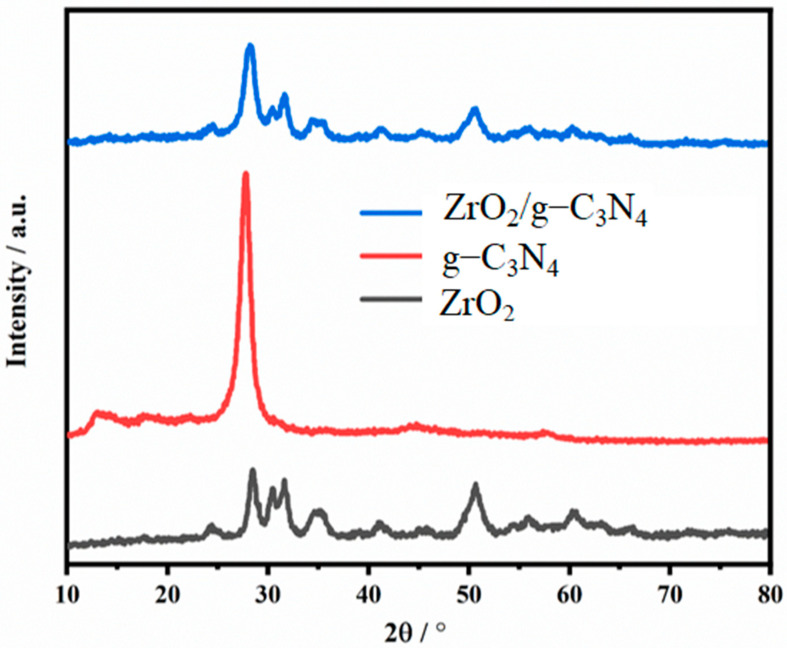
XRD patterns of polymeric ZrO_2_/g-C_3_N_4_ nanocomposite, pure g-C_3_N_4_ nanosheet, and pure ZrO_2_ nanoparticles.

Using an X-ray powder diffraction (XRD) analysis, the phase structures of the ZrO_2_, g-C_3_N_4_, and ZrO_2_/g-C_3_N_4_ nanocomposites were investigated. The XRD spectra of the samples are displayed in Figure 4. The (100) and (002) planes of the graphitic carbon nitride phase were indicated by the two diffraction peaks in the g-C_3_N_4_ sheet at 12.9° and 27.7°, respectively. These planes were fitted with units of layered and tri-s-triazine [39,40]. According to the results, the produced ZrO_2_ nanoparticles exhibited both monoclinic (JCPDS 07–0343) and tetragonal (JCPDS 80–2155) phases. The monoclinic phase is easily given to planes (011), (111), (−111), (022), (211), (−112), (−202), and (013), respectively. The diffraction peaks at 2θ correspond to the following: 24.35°, 28.4°, 31.6°, 34.6°, 40.95°, 45.75°, 54.1°, and 55.7°. The tetragonal phase is confirmed by the peaks at 30.4°, 35.13°, 50.475°, and 60.07° 2θ, which correspond to the [101], [110], [112], and [211] planes. Nonetheless, the ZrO_2_ nanoparticles’ predominant crystalline structure is the monoclinic phase [38]. The ZrO_2_/g-C_3_N_4_ nanocomposite’s diffraction peak at approximately 28.2° may be the result of an overlap between the ZrO_2_ ((−111) plane) and g-C_3_N_4_ ((002) plane) reflection peaks. The assigned ZrO_2_ nanostructure planes provide additional proof of the ZrO_2_/g-C_3_N_4_ nanocomposite’s successful synthesis.

**Figure 5:**
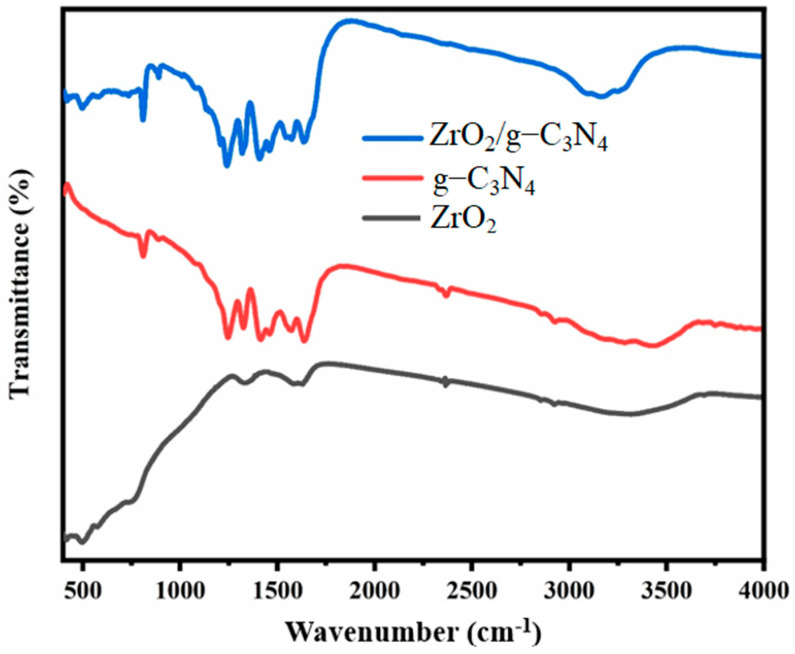
FTIR spectra of pure ZrO_2_ nanoparticles, pure g-C_3_N_4_ nanosheets, and ZrO_2_/g-C_3_N_4_ nanocomposite.

To have a better understanding of the chemical makeup of both the pure components and their nanocomposites, a Fourier transform infrared spectrometer (FTIR) analysis was employed, as displayed in Figure 5. According to the g-C_3_N_4_-associated FTIR spectra, the 808.02 cm^−1^ sharp peak is caused by a tri-s-triazine vibration bending unit. Furthermore, a broad peak in the 3000–3400 cm^−1^ range and a series of distinct peaks in the 1000–1700 cm^−1^ range originate from the primary and secondary amine vibration modes [“N-H” and “NH_2_”] and the g-C_3_N_4_ heterocycle stretching modes [“C-N” and “C=N”], respectively [41,42]. The ZrO_2_/g-C_3_N_4_ nanocomposite sample’s FTIR spectrum resembles that of a pure g-C_3_N_4_ nanosheet, as seen in Figure 5. Nonetheless, the existence of peaks at 500 and 3200 cm^−1^ suggests that ZrO_2_ nanoparticles are present in the nanocomposite sample. Based on the FTIR study results, the ZrO_2_/g-C_3_N_4_ nanocomposite’s chemical structure remained unchanged after compositing in comparison to the pure components.

**Figure 6:**
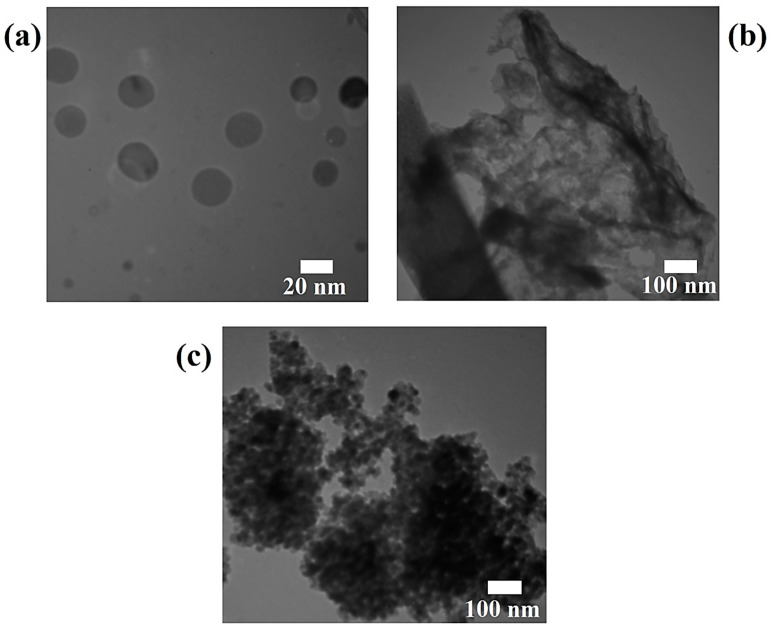
TEM images of (**a**) pure ZrO_2_ nanoparticles, (**b**) pure g-C_3_N_4_ nanosheet, and (**c**) ZrO_2_/g-C_3_N_4_ nanocomposite.

A transmission electron microscopy (TEM) analysis is used to morphologically analyze pure materials and nanocomposite materials in order to determine their shape, size, and structural properties. Figure 6 displays the ZrO_2_ nanoparticles’ TEM picture. The produced ZrO_2_ is spherical, with an average nanosphere diameter of roughly 20 nm, as can be seen visually. The g-C_3_N_4_ nanosheet’s TEM picture is displayed in Figure 6b. The thermal exfoliation of the g-C_3_N_4_ bulk results in the sizable, ultrathin, wrinkled nanosheet shape that is clearly seen in pure g-C_3_N_4_. The ZrO_2_ nanoparticles are placed on g-C_3_N_4_ nanosheets to create a ZrO_2_/g-C_3_N_4_ nanocomposite, as shown in Figure 6c. The ZrO_2_ nanoparticles in the nanocomposite sample resemble pure ZrO_2_ in both size and form. On the surface of the g-C_3_N_4_ nanosheet, ZrO_2_ nanoparticles are equally and evenly distributed, as shown in Figure 6c.

**Figure 7:**
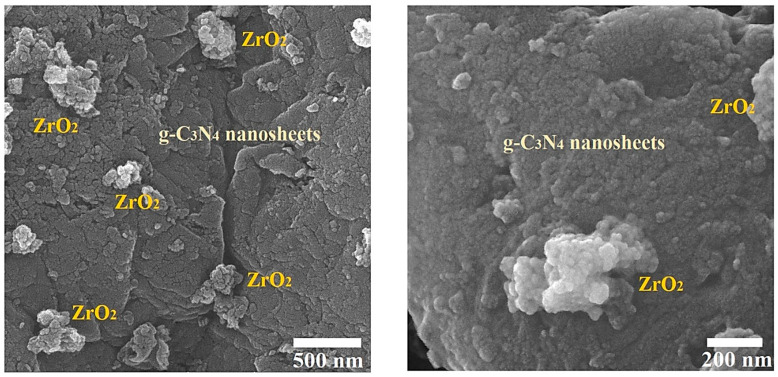
FESEM images of polymeric ZrO_2_/g-C_3_N_4_ nanocomposite with various magnifications.

Another field emission scanning electron microscope (FESEM) analysis was used to characterize the morphological examination in order to learn more about the ZrO_2_/g-C_3_N_4_ nanocomposite’s surface. As can be seen in Figure 7, the ZrO_2_ nanoparticles are uniformly dispersed and have good interaction with g-C_3_N_4_ on the material’s broad nanosheet surface.

**Figure 8:**
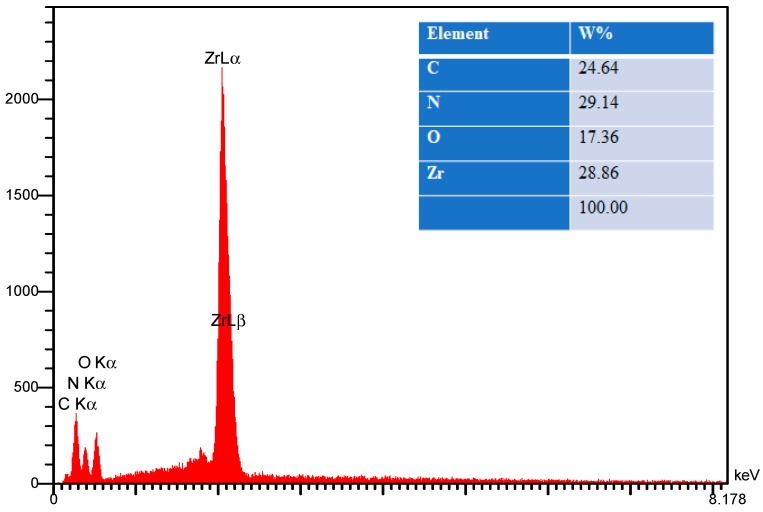
EDX image of polymeric ZrO_2_/g-C_3_N_4_ nanocomposite.

To confirm that the nanocomposite seen in Figure 6c belongs to both g-C_3_N_4_ and ZrO_2_, an energy-dispersive X-ray testing (EDX) analysis was performed (Figure 8). ZrO_2_ and g-C_3_N_4_ in the produced nanocomposite are confirmed by the presence of components such as carbon (C), nitrogen (N), oxygen (O), and zirconium (Zr).

**Figure 9:**
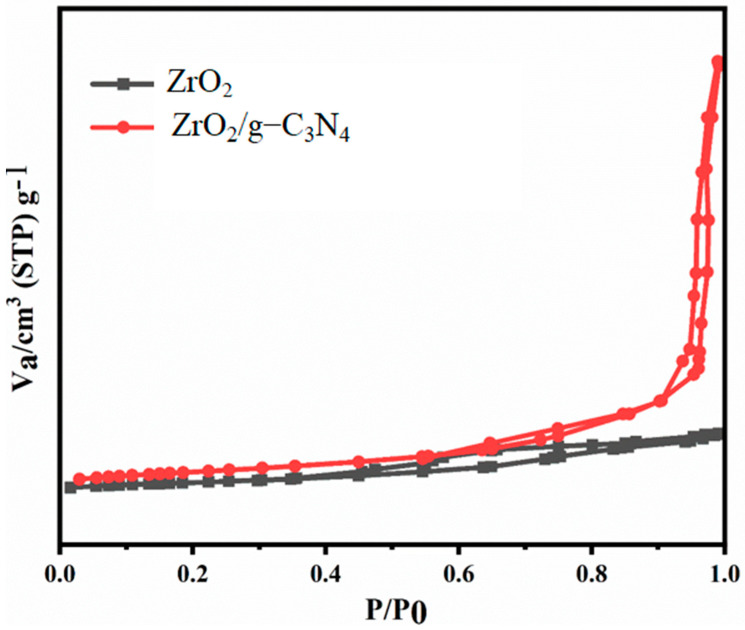
N_2_ adsorption–desorption isotherms of pure ZrO_2_ nanoparticles and polymeric ZrO_2_/g-C_3_N_4_ nanocomposite.

The textural properties of the produced nanocomposite and pure ZrO_2_ were examined using the Brunauer–Emmett–Teller (BET) analysis. The N_2_ adsorption–desorption isotherms for the ZrO_2_ and ZrO_2_/g-C_3_N_4_ nanocomposite are shown in Figure 9. Both diagrams demonstrate the existence of mesopores and micropores in the samples as-prepared by the type IV isotherm with a loop of H-type (hysteresis loop of H_3_-type) [41]. As shown, the ZrO_2_/g-C_3_N_4_ nanocomposite’s specific surface area increased to 88.16 m^2^/g from 49.27 m^2^/g for pure ZrO_2_. Additionally, when compared to pure ZrO_2_ nanoparticles, the ZrO_2_/g-C_3_N_4_ nanocomposite has a total pore volume that is approximately seven times larger (Table 1).

**Figure 10:**
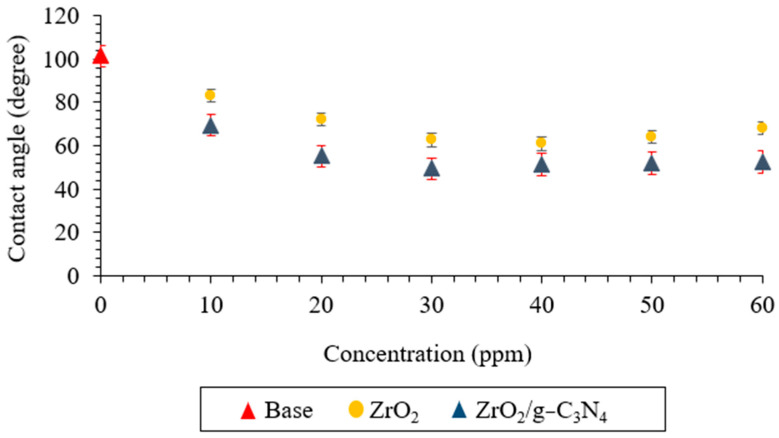
Contact angles using ZrO_2_ and polymeric ZrO_2_/g-C_3_N_4_ nanomaterials for obtaining optimum concentration.

An additional, significant element influencing oil output is wettability alteration (WA). WA, a crucial water displacement factor, was changed by the nanoparticles and improved the minerals’ ability to recover oil. Fluid–rock interactions are changed by nanofluid, and the recovery factor was raised by employing nanocomposites because of the effective modification of the IFT and CA. The effect of the ZrO_2_ and ZrO_2_/g-C_3_N_4_ nanomaterial concentrations on the contact angle is depicted in Figure 10. The contact angle with base fluid (no nanoparticles) is 101.43°. At 3800 psi pressure and 78 °C, both ZrO_2_ and ZrO_2_/g-C_3_N_4_ nanofluids improve the carbonate hydrophilicity by decreasing the contact angle. The lowest contact angles, 49.49° (at 30 ppm) and 61.00° (at 40 ppm), were achieved with the ZrO_2_ and ZrO_2_/g-C_3_N_4_ nanomaterials, respectively. This could be a result of the ZrO_2_/g-C_3_N_4_ nanocomposites enhancing the hydrophilic properties of the carbonate rock. According to Rostami et al., the van der Waals force is the primary factor influencing wettability [43].

**Figure 11:**
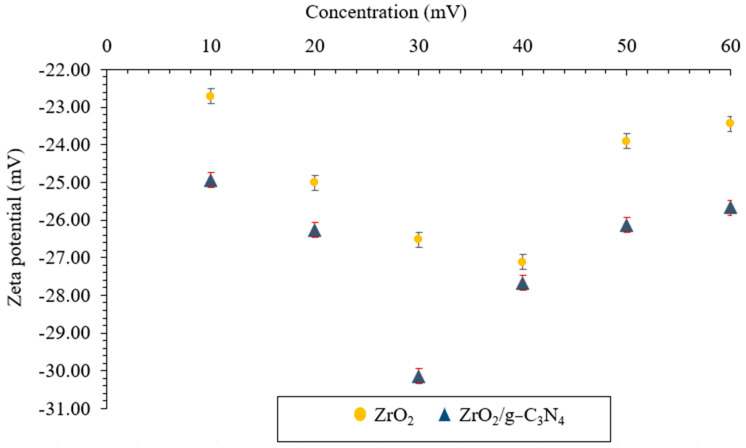
Zeta potentials using ZrO_2_ and polymeric ZrO_2_/g-C_3_N_4_ nanomaterials.

The effect of base fluid ZrO_2_ and ZrO_2_/g-C_3_N_4_ nanomaterials concentrations on the zeta potential is depicted in Figure 11. This test was conducted using the Malvern Zetasizer Nano ZS ZEN3600 (Worcestershire, UK). This experiment demonstrates that nanofluids are significantly stabilized by the addition of ZrO_2_ (40 ppm) and ZrO_2_/g-C_3_N_4_ (30 ppm) nanomaterial concentrations. The ZrO_2_/g-C_3_N_4_ nanocomposite showed fully stable conditions at 30 ppm, with zeta potentials of −30.14 mV.

**Figure 12:**
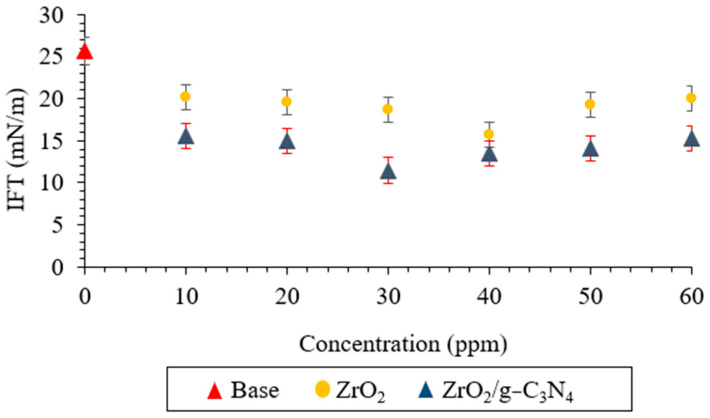
Interfacial tension measurements using ZrO_2_ and polymeric ZrO_2_/g-C_3_N_4_ nanomaterials.

Figure 12 illustrates how the presence of ZrO_2_ and ZrO_2_/g-C_3_N_4_ nanomaterials influences the crude oil–water IFT at 3800 psi pressure and 78 °C. This figure illustrates how ZrO_2_ and ZrO_2_/g-C_3_N_4_ nanofluids reduced the base fluid’s IFT. The base fluid’s initial IFT decreased from 25.68 mN/m to 11.47 mN/m (at 30 ppm) and 15.9 mN/m (at 40 ppm) upon the addition of the ZrO_2_ and ZrO_2_/g-C_3_N_4_ nanofluids, respectively.

**Figure 13:**
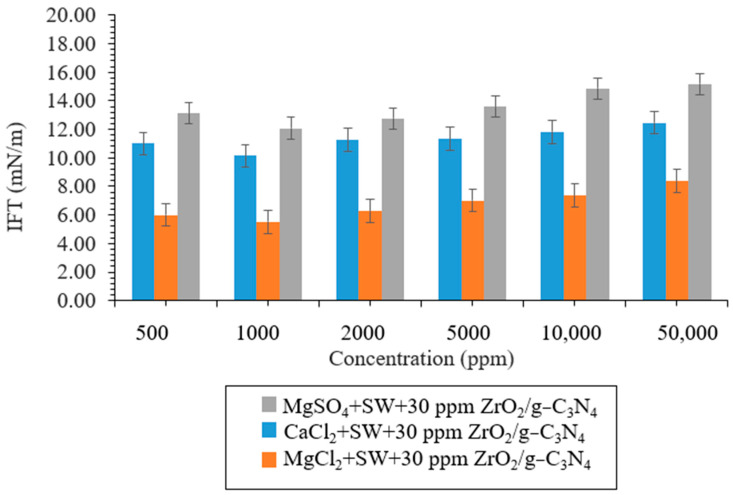
An evaluation of the IFT in the presence of 40 ppm polymeric nanocomposites and different brine concentrations.

According to the IFT, CA, and zeta potential results, the optimum concentrations for the ZrO_2_ and ZrO_2_/g-C_3_N_4_ nanomaterials were 40 and 30 ppm, respectively. Moreover, the ZrO_2_/g-C_3_N_4_ nanocomposite showed better results in the obtained EOR parameters, and it was selected for performing the LSWF at 30 ppm. As seen in Figure 13, a number of IFT tests were conducted for optimal brine solutions employing various ions, such as CaCl_2_ + SW + 30 ppm ZrO_2_/g-C_3_N_4_, MgCl_2_ + SW + 30 ppm ZrO_2_/g-C_3_N_4_, and MgSO_4_ + SW + 30 ppm ZrO_2_/g-C_3_N_4_, at various concentrations, including 500, 1000, 2000, 5000, 10,000, and 50,000 ppm. CaCl_2_ + SW + 30 ppm ZrO_2_/g-C_3_N_4_, MgCl_2_ + SW + 30 ppm ZrO_2_/g-C_3_N_4_, and MgSO_4_ + SW + 30 ppm ZrO_2_/g-C_3_N_4_ had minimal IFTs at 1000 ppm, according to the results. The order of the IFT decrease was as follows: MgCl_2_ + SW + 30 ppm ZrO_2_/g-C_3_N_4_ > CaCl_2_+ SW + 30 ppm ZrO_2_/g-C_3_N_4_ > MgSO_4_+ SW + 30 ppm ZrO_2_/g-C_3_N_4_. The identical sequence for the contact angle decrease is displayed in Table 2 and reflects the following: MgCl_2_ + SW + 30 ppm ZrO_2_/g-C_3_N_4_ > CaCl_2_+ SW + 30 ppm ZrO_2_/g-C_3_N_4_ > MgSO_4_+ SW + 30 ppm ZrO_2_/g-C_3_N_4._. The presence of MgCl_2_ + SW + 30 ppm ZrO_2_/g-C_3_N_4_, CaCl_2_ + SW + 30 ppm ZrO_2_/g-C_3_N_4_, and MgSO_4_ + SW + 30 ppm ZrO_2_/g-C_3_N_4_ reduced the base contact angle from 101.43° to 38.12°, 50.50°, and 68.33°. Furthermore, all of the created solutions have stable conditions, according to the ZP findings [44]. The ZP is −31.25 mV for MgCl_2_+ SW + 30 ppm ZrO_2_/g-C_3_N_4_, −30.12 mV for CaCl2+ SW + 30 ppm ZrO_2_/g-C_3_N_4_, and −28.89 mV for MgSO_4_+ SW + 30 ppm ZrO_2_/g-C_3_N_4_. The findings of the zeta potential, contact angle, and interfacial tension are consistent with those of Lashingkarbolooki et al. (2018) [45]. The Malvern Zetasizer Nano ZS ZEN3600 (Worcestershire, UK) was used in this study to analyze the particle size distributions of the zeolite-zirconia-cerium oxide nanocomposites in the prepared nanofluids.

**Figure 14:**
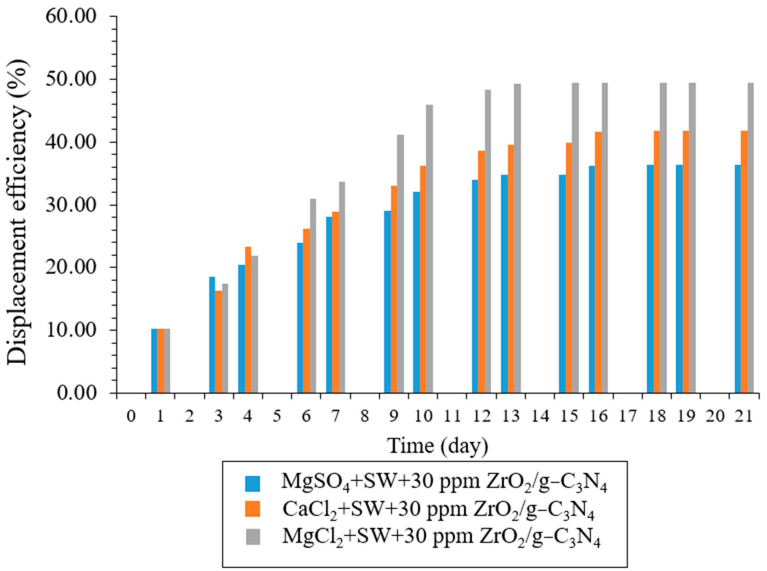
The recovery factor using 30 ppm polymeric nanocomposites and different brine concentrations.

To observe the designed solutions’ effects in the carbonate reservoir, series imbibition tests were conducted at 1000 ppm of each brine, including CaCl_2_ + SW, MgCl_2_ + SW, and MgSO_4_ + SW, as well as 30 ppm of ZrO_2_/g-C_3_N_4_. We carried out the spontaneous imbibition testing at 70 °C and 3200 psi pressure, much like in the earlier IFT and contact angle tests. As illustrated in Figure 14, the recovery factor increases in the following manner (in order): MgCl_2_ + SW > CaCl_2_ + SW > MgSO_4_ + SW at 1000 ppm, 78 °C, 3800 psi, and 30 ppm of ZrO_2_/g-C_3_N_4_. MgCl_2_ + SW + 30 ppm ZrO_2_/g-C_3_N_4_, CaCl_2_+ SW + 30 ppm ZrO_2_/g-C_3_N_4_, and MgSO_4_ + SW + 30 ppm ZrO_2_/g-C_3_N_4_ yielded the final recovery factors of 49.39%, 41.85%, and 36.32%, respectively, during a 21-day test. The primary causes of the ZrO_2_/g-C_3_N_4_ nanofluid flooding’s increased oil recovery include the WA, IFT, and ZP alterations. Furthermore, in accordance with earlier IFT, CA, and ZP studies, MgCl_2_ + SW + 30 ppm ZrO_2_/g-C_3_N_4_ nanofluids consistently recover more oil than CaCl_2_ + SW + 30 ppm ZrO_2_/g-C_3_N_4_ and MgSO_4_ + SW + 30 ppm ZrO_2_/g-C_3_N_4_.

Table 3 compares the results of this study with other research findings. Based on the results, MgCl_2_ + SW + 30 ppm, and even other salinities in this study, demonstrated an excellent recovery in carbonate porous media.

## 4. Conclusions

Novel ZrO_2_/g-C_3_N_4_ nanocomposites were applied in this lab-scale investigation to increase the effectiveness of low salinity flooding in carbonate porous media at 78 °C and 3800 psi, and the results were compared with pure ZrO_2_ nanoparticles. The primary findings were as follows:

- The ZrO_2_/g-C_3_N_4_ nanocomposite’s specific surface area increased to 88.16 m^2^/g from 49.27 m^2^/g for the pure ZrO_2_.
- The optimal concentrations for the ZrO_2_ and ZrO_2_/g-C_3_N_4_ nanomaterials were 40 and 30 ppm, respectively, based on the IFT, CA, and zeta potential findings (among several tested concentrations, which ranged from 10 to 60 ppm).
- The IFT, zeta potential, and CA were (11.47 mN/m, −30.14 mV, and 49.49° at 30 ppm), (15.69 mN/m, −27.12 mV, and 61.00° at 40 ppm) for the ZrO_2_/g-C_3_N_4_ and ZrO_2_, respectively.
- The ZrO_2_/g-C_3_N_4_ nanocomposites were chosen to perform the LSWF at 30 ppm due to their superior performance in the obtained EOR parameters.
- The results showed the lowest interfacial tensions at 1000 ppm for CaCl_2_ + SW + 30 ppm ZrO_2_/g-C_3_N_4_, MgCl_2_ + SW + 30 ppm ZrO_2_/g-C_3_N_4_, and MgSO_4_ + SW + 30 ppm ZrO_2_/g-C_3_N_4_, and the interfacial tension and contact angle followed the order as follows: MgCl_2_ + SW + 30 ppm ZrO_2_/g-C_3_N_4_> CaCl_2_+ SW + 30 ppm ZrO_2_/g-C_3_N_4_ > MgSO_4_ + SW + 30 ppm ZrO_2_/g-C_3_N_4_.
- The MgCl_2_ + SW + 30 ppm ZrO_2_/g-C_3_N_4_ had the highest stability of any of the brine solutions, measuring −31.25 mV at 1000 ppm.
- The recovery factor following a 21-day test was 49.39% for MgCl_2_ + SW + 30 ppm ZrO_2_/g-C_3_N_4_, 41.85% for CaCl_2_ + SW + 30 ppm ZrO_2_/g-C_3_N_4_, and 36.32% for MgSO_4_ + 10 SW + 30 ppm ZrO_2_/g-C_3_N_4_.

## Figures and Tables

**Table 1:** ZrO_2_ and polymeric ZrO_2_/g-C_3_N_4_ nanocomposite surface area, total pore volume, and pore diameter.

Sample	Surface Area (m^2^/g)	Pore Diameter (nm)	Total Pore Volume (cm^3^/g)
ZrO_2_	49.27	7.99	0.0985
ZrO_2_/g-C_3_N_4_ nanocomposite	88.16	30.99	0.6832

**Table 2:** The zeta potentials and contact angles in the presence of 40 ppm polymeric nanocomposites and 2000 ppm of different brine concentrations.

Salt Type	Salt Concentration (ppm)	ZrO_2_/g-C_3_N_4_ Concentration (ppm)	ZP (mV)	CA (°)
CaCl_2_ + SW	1000	30	−30.12	50.50
MgCl_2_ + SW	1000	30	−31.25	38.12
MgSO_4_ + SW	1000	30	−28.89	68.33

**Table 3:** Comparing current study with other research.

Nanoparticles	Permeability (mD)	Oil Recovery (%)	References
MgCl_2_ + SW + 30 ppm ZrO_2_/g-C_3_N_4_	10.78	49.39	Current study
CaCl_2_+ SW + 30 ppm ZrO_2_/g-C_3_N_4_	10.78	41.85	Current study
MgSO_4_ + SW + 30 ppm ZrO_2_/g-C_3_N_4_	10.78	36.32	Current study
Polymeric ZnO/SiO_2_	10.45	34.10	[46]
Silica	5.00	16.00	[47]
Deionized/Alumina	35.99	43.81	[48]
Silica	0.21	16.00	[49]
Zinc oxide	0.309	8.89	[50]
Gamma-Alumina	46–68	11.50	[51]

## Data Availability

The original contributions presented in this study are included in the article. Further inquiries can be directed to the corresponding authors.
